# Rhein ameliorates MASH via EGFR/AKT/PPARα-mediated coordinated regulation of metabolism and inflammation

**DOI:** 10.3389/fphar.2026.1844294

**Published:** 2026-06-23

**Authors:** Liyan Song, Shuang Hua, Qin Zeng, Yingqiang Fu, Ziqi Sui

**Affiliations:** 1 Department of Gastroenterology, The First People’s Hospital of Linping District, Hangzhou, Zhejiang, China; 2 Department of Cardiology, The Second Affiliated Hospital of Zhejiang Chinese Medical University, Hangzhou, Zhejiang, China; 3 Department of Gastroenterology, The Second Affiliated Hospital of Zhejiang Chinese Medical University, Hangzhou, China; 4 Department of Thyroid and Breast Surgery, The First People’s Hospital of Linping District, Hangzhou, Zhejiang, China

**Keywords:** lipid metabolism, metabolic dysfunction-associated steatohepatitis (MASH), network pharmacology, PI3K/akt signaling pathway, Rhein

## Abstract

**Background:**

Metabolic dysfunction-associated steatohepatitis (MASH) presents a growing global health challenge with limited therapeutic options. Rhein, an active anthraquinone derived from the traditional medicine rhubarb (*Rheum palmatum L.*), has demonstrated potential in alleviating metabolic liver disorders, yet its precise mechanism of action against MASH remains unclear.

**Methods:**

An integrated strategy combining network pharmacology, molecular docking, and experimental validation was employed. A “Rhein-MASH” target network was constructed to identify core targets. The *in vivo* therapeutic effects and dose dependency of Rhein were assessed in a choline-deficient, high-fat diet (CDAHFD)-induced mouse MASH model. *In vitro* mechanisms were investigated in PA/OA-treated AML12 hepatocytes using functional assays, pharmacological inhibitors, and siRNA-mediated gene knockdown. Key signaling molecules and gene expression were analyzed via Western blot, qPCR, and immunohistochemistry.

**Results:**

Rhein treatment improved hepatic steatosis, inflammation, and liver injury in MASH mice in a dose-dependent manner without altering systemic lipid profiles. It selectively upregulated the expression of key genes involved in fatty acid β-oxidation (*Acadl*, *Cpt1a*) while suppressing pro-inflammatory cytokines (*Tnf-α*, *Il-1β*). Network pharmacology and molecular docking identified EGFR as a core target, with Rhein demonstrating potent binding affinity (docking score: −7.9 kcal/mol). *In vitro* experiments revealed that Rhein acts through EGFR to induces its autophosphorylation, and sequentially activates the downstream PI3K/AKT signaling pathway. This signaling module coordinately enhances fatty acid oxidation–related gene expression and reduces inflammatory gene expression partly through PPARα, thereby effectively ameliorating lipid accumulation and inflammatory phenotypes in hepatocytes.

**Conclusion:**

This study elucidates that Rhein ameliorates MASH by activating the EGFR/PI3K/AKT/PPARα pathway, which coordinately enhances hepatic fatty acid oxidation and suppresses inflammation. These findings position Rhein as a multi-target regulator and suggest the EGFR-PPARα axis is a promising therapeutic target for MASH.

## Introduction

Metabolic dysfunction-associated steatotic liver disease (MASLD) is a complex, spectrum liver disease of global epidemic proportion, paralleling the rise in obesity and type 2 diabetes (Do et al., 2025; Kan et al., 2025; Steinberg et al., 2025a). It currently affects approximately 30% of adults worldwide, representing a leading cause of liver-related morbidity and mortality. Its progressive form, metabolic dysfunction-associated steatohepatitis (MASH), develops in about 20%–30% of MASLD patients (Younossi et al., 2025). Histologically, MASH is defined by the presence of >5% hepatic steatosis, hepatocellular ballooning, and lobular inflammation, features that collectively confer a significantly elevated risk for fibrosis, cirrhosis, and hepatocellular carcinoma (Kleiner et al., 2005; Kleiner and Brunt, 2012; Farrell et al., 2019). Notably, liver-related mortality in MASH is over six-fold higher than in simple steatosis, highlighting its substantial clinical burden. The core pathogenesis of MASH originates from excessive hepatocellular lipid accumulation (Do et al., 2025; Steinberg et al., 2025b). This steatosis is not inert; the resultant lipotoxic stress—manifested by oxidative stress, endoplasmic reticulum stress, and mitochondrial dysfunction—directly induces hepatocyte injury and apoptosis (Wang et al., 2025). The release of damage-associated molecular patterns from injured cells initiates and sustains a potent inflammatory cascade (He et al., 2025). This chronic inflammatory milieu, rich in pro-fibrogenic cytokines, potently activates hepatic stellate cells (HSCs), leading to excessive extracellular matrix deposition and fibrosis progression (Kisseleva et al., 2025; Zhang et al., 2025). Collectively, these interconnected processes form a self-perpetuating vicious cycle of steatosis, lipotoxicity, inflammation, and fibrosis, which constitutes the central pathogenic axis driving MASH toward end-stage liver disease.

Rhein, a naturally occurring anthraquinone compound isolated from the traditional medicinal plant rhubarb (*Rheum palmatum L.*) (Zhou et al., 2015), has emerged in recent years as a promising candidate for the treatment of MASH (Qian F. et al., 2024; Zhao et al., 2024; Dai et al., 2025; Yuan et al., 2025). In traditional medicine, herbal preparations containing Rhein have been widely used for a variety of purposes, including anti-inflammatory, antibacterial, antiviral, detoxifying, antipyretic, and laxative effects (Wang et al., 2020; Cheng et al., 2021; Feng et al., 2025). Modern pharmacological studies have shown that Rhein possesses not only broad-spectrum biological activities but also exhibits significant effects in improving lipid metabolism disorders and suppressing inflammatory responses (Cheng et al., 2021; El-Refaie et al., 2023). In MASH models, Rhein and its derivatives (e.g., Rhein Lysinate) effectively reduce hepatic lipid accumulation, downregulate the expression of inflammatory factors, and improve liver function (Dai et al., 2025; Yuan et al., 2025). Its mechanisms involve multiple signaling networks, including -mediated regulation of lipid metabolism, the AMPK/ACC pathway-modulated cellular energy balance, and the axis-mediated regulation of macrophage polarization (Sheng et al., 2011; Fu et al., 2024; Yuan et al., 2025).

However, within the complex pathological microenvironment of MASH, it remains unclear whether there exists a common upstream regulatory node among these signaling pathways, and whether Rhein can integrate its dual effects on metabolic regulation and anti-inflammation via a dominant signaling axis. Therefore, this study aims to systematically elucidate the molecular mechanism by which Rhein ameliorates MASH through the EGFR/AKT/PPARα signaling axis, employing a strategy combining network pharmacology and experimental biology. First, a “Rhein-MASH” target regulatory network will be constructed by integrating databases such as TCMSP, GeneCards, and DisGeNET using network pharmacology approaches. Core targets EGFR will be screened, and the binding mode and affinity between Rhein and the EGFR protein will be validated by molecular docking simulations. Subsequently, *in vitro* and *in vivo* MASH models, techniques such as Western blot, qPCR, and immunofluorescence will be utilized to examine the effects of Rhein on EGFR phosphorylation, AKT activation, and PPARα nuclear translocation. By measuring the activity and expression of key enzymes involved in fatty acid oxidation (*Acadl*, *Cpt1a*) and the expression levels of inflammatory cytokines (*TNF-α*, *IL-6*), the role of this pathway in promoting oxidative decomposition and inhibiting inflammation will be clarified. Finally, pharmacological reverse validation will be performed using siRNA-mediated EGFR knockdown, AKT inhibitors, and PPARα agonists to confirm the necessity of this signaling axis in the amelioration of MASH by Rhein.

## Methods

### Animals and experimental design

The animal study was approved by the Animal Ethics Committee of the Second Affiliated Hospital of Zhejiang Chinese Medical University (approval No. IACUC-20260413-15). The study was conducted in accordance with the local legislation and institutional requirements. Eight-week-old male C57BL/6J mice, purchased from GemPharmatech Co., Ltd. (Suzhou, China), were used in the experiments and housed under specific pathogen-free conditions with controlled temperature and humidity, a 12-h light–dark cycle, and *ad libitum* access to food and water.

### Experiment 1: dose-dependent intervention of Rhein on MASH phenotypes

To systematically evaluate the interventional effect of Rhein on metabolic dysfunction-associated steatohepatitis (MASH) and elucidate its dose dependency, we established a mouse MASH model induced by feeding a choline-deficient, high-fat diet (CDAHF60, 60% kcal from fat, Research Diets Inc.). Mice were randomly divided into five groups (n = 5 per group): (1) Normal control group (Healthy), fed a standard chow diet throughout the experiment; (2) MASH model group (Model), fed the CDAHF60 diet and administered the solvent vehicle via daily oral gavage; (3–5) Rhein intervention groups, fed the CDAHF60 diet and, after 4 weeks of diet induction, receiving daily oral gavage of Rhein at low (20 mg/kg/day) (Low-Rhein), medium (50 mg/kg/day) (Medium-Rhein), or high (120 mg/kg/day) doses (High-Rhein). All groups fed the CDAHF60 diet continued on this diet until the experimental endpoint. The intervention period lasted for 8 weeks, resulting in a total experimental duration of 12 weeks.

### Experiment 2: mechanism validation study based on EGFR knockdown

To verify *in vivo* whether Rhein exerts its therapeutic effects by acting on EGFR, we established a hepatocyte-specific EGFR knockdown mouse model and designed the following five experimental groups (n = 5 per group): (1) Normal control group (Healthy), receiving tail vein injection of the control virus AAV8-scramble and fed a standard chow diet throughout the experiment; (2) MASH model group, receiving AAV8-scramble via tail vein injection and fed the CDAHF60 diet (Model); (3) Rhein treatment group (Rhein), injected with AAV8-scramble and fed the CDAHF60 diet for 4 weeks, followed by daily oral gavage of Rhein (120 mg/kg/day) for an additional 8 weeks; (4) EGFR knockdown group (sh*Egfr*), receiving tail vein injection of AAV8-shEGFR to knock down hepatocyte EGFR expression and fed the CDAHF60 diet; (5) EGFR knockdown + Rhein treatment group (sh*Egfr*-Rhein), injected with AAV8-shEGFR and fed the CDAHF60 diet for 4 weeks, followed by daily oral gavage of Rhein (120 mg/kg/day) for 8 weeks.

### Cell culture and transfection

The AML12 (alpha mouse liver 12) immortalized mouse hepatocyte line was purchased from the National Collection of Animal Cell Cultures (#GNM42, NCACC, China). Cells were cultured in dulbecco’s modified eagle’s medium/nutrient mixture F-12 (DMEM/F-12, # G4612, Servicebio, Wuhan, China) supplemented with 10% fetal bovine serum (# G8003, Servicebio, Wuhan, China), 1% Insulin-Transferrin-Selenium (ITS, # 41400045, Thermo Fisher, United States), 40 ng/mL dexamethasone (# HY-14648, MedChemExpress, United States), and 1% penicillin-streptomycin (# G4016, Servicebio, Wuhan, China). Cells were maintained at 37 °C in a humidified incubator with 5% CO_2_.

siRNA transfection was performed using a lipid-based method. *Egfr* or scramble siRNA (The sequences are provided in the Supplementary Table S1) was diluted in serum-free medium to a working concentration and mixed with an equal volume of diluted Lipofectamine™ RNAiMAX reagent. After incubating for 15 min at room temperature to form transfection complexes, the mixture was added to AML12 cells at 60% confluence, which were maintained in antibiotic-free DMEM/F-12 medium supplemented with 10% FBS. The transfection medium was replaced with fresh complete medium after 6–8 h, and cells were cultured for 48 h before harvesting. Knockdown efficiency was assessed by qRT-PCR or Western blot analysis.

### Construction and administration of AAV-shEGFR

For hepatocyte-specific knockdown of EGFR, an AAV8 vector expressing shRNA targeting mouse *Egfr* was constructed based on the NCBI RefSeq NM_007912.4. A scrambled shRNA was used as control (The sequences are provided in the Supplementary Table S1). Recombinant AAV8 particles (AAV8-shEGFR or AAV8-scramble) were packaged, purified, and titered by Sangon Biotech (Shanghai, China) using a triple-plasmid transfection system, with viral genome titer adjusted to 1.0×10^13^ vg/mL. Mice received a single tail-vein injection of 1 × 10^11^ vg virus in 100 μL sterile PBS.

### Biochemical analysis

Serum lipid profiles and liver enzyme activities were assessed using commercial assay kits according to the manufacturers’ protocols. Briefly, serum levels of triglycerides (TG) and total cholesterol (TC) were quantified using kits from Solarbio (Beijing, China). Serum alanine aminotransferase (ALT) and aspartate aminotransferase (AST) activities were determined using kits purchased from the Nanjing Jiancheng Bioengineering Institute (Nanjing, China).

## Extraction of triglycerides from liver tissue

Hepatic triglyceride (TG) content was extracted using a modified organic solvent method. Briefly, approximately 100 mg of frozen liver tissue was homogenized in 500 μL of ice-cold PBS. Total lipids were then extracted by adding 4 mL of chloroform/methanol (2:1, v/v) to the homogenate. After adding 2 mL of 0.6% NaCl and vortexing, the mixture was centrifuged at 2,000 × g for 20 min at 4 °C. The lower organic phase was collected and dried. The lipid pellet was dissolved in 100 μL of PBS containing 1% Triton X-100. Finally, the TG concentration in the extract was measured immediately using a commercial enzymatic assay kit, and the values were normalized to the tissue protein content.

### Quantitative real-time PCR (qRT-PCR)

Total RNA was extracted with RNAiso Plus (#9109, TakaRa Bio Inc., Shiga, Japan) as previously described. RNA was reverse transcribed into cDNA with PrimeScript RT reagent Kit (#RR037, TakaRa Bio Inc.). cDNA was then amplified with SYBR®Premix Ex Taq™ (#RR041, TakaRa Bio Inc., Shiga, Japan). The real-time PCR was conducted with a LightCycler 96 RT-qPCR System (Roche, Basel, Switzerland). The relative quantity of the targeted RNA was calculated through normalization to the quantity of the corresponding β-actin mRNA level. Detailed primer sequences are listed in Supplementary Table S1.

### Western blot

Total protein was extracted from both cultured cells and liver tissues. Cells were lysed on ice using RIPA buffer (# G2002, Servicebio, Wuhan, China) containing protease and phosphatase inhibitors. Liver tissue (∼30 mg) was homogenized in the same lysis buffer. Lysates were centrifuged at 12,000 × g for 15 min at 4 °C to collect the supernatant. Protein concentration was determined using a BCA assay kit (# G2026, Servicebio, Wuhan, China). For immunoblotting, equal amounts of protein were separated by SDS-PAGE and transferred to PVDF membranes. After blocking with 5% non-fat milk, membranes were incubated overnight at 4 °C with primary antibodies, followed by incubation with HRP-conjugated secondary antibodies (The antibodies are provided in the Supplementary Table S2). Protein bands were visualized using an ECL chemiluminescent reagent (# G2014, Servicebio, China) and imaged.

## Histopathological examination and immunohistochemistry

Liver tissues were fixed in 4% paraformaldehyde, embedded in paraffin, and sectioned at 4 μm thickness. For histopathological evaluation, sections were stained with Hematoxylin and Eosin (H&E) to assess steatosis, inflammation, and ballooning. Hepatic steatosis, inflammation, and ballooning were assessed semiquantitatively by one pathologist, and the results were independently verified by a second pathologist. Both were blinded to the experimental groups. The following scoring criteria were used based on the NASH Clinical Research Network scoring system (Kleiner et al., 2005).

For immunohistochemistry, antigen retrieval was performed, followed by blocking with serum. Sections were incubated overnight at 4 °C with F4/80 primary antibodies, then with appropriate secondary antibodies, and developed with DAB substrate. The F4/80-positive area was quantified using ImageJ software in five randomly selected fields per section. All slides were imaged under a light microscope.

### Oil red O staining

Cells were washed once with ice-cold PBS and fixed with 4% paraformaldehyde for 15 min. The Oil Red O working solution was freshly prepared by mixing stock solution with distilled water at a 3:2 ratio, equilibrated for 10 min, and filtered through a 0.45 µm filter. After fixation, cells were washed twice with PBS and treated with 60% isopropanol for 5 min. The isopropanol was removed and cells were incubated with the filtered Oil Red O working solution for 20 min. Finally, the staining solution was removed, cells were washed twice with PBS, and images were captured under an inverted microscope.

### CCK-8 assays

Cells were seeded into 96-well plates at a density of 4 × 10^3^ cells per well and allowed to adhere overnight. To assess the direct effect of Rhein, cells were treated with a gradient of Rhein concentrations (0, 5, 10, 20, 40, 80,120 µM) for a specified duration. To evaluate the protective effect of Rhein against lipotoxicity, cells were first treated with a palmitic acid/oleic acid (PA/OA) mixture (400 μM PA: 800 µM OA at a 2:1 M ratio) for 24 h to induce lipid overload and injury, followed by co-treatment with the same gradient of Rhein concentrations for another 24 h. Following the respective treatments, 10 µL of CCK-8 reagent was added to each well, and the plates were incubated at 37 °C for 1 h. The absorbance at 450 nm was measured using a microplate reader.

### Network pharmacology analysis and molecular docking

Potential targets of Rhein were predicted using the SwissTargetPrediction database (http://www.swisstargetprediction.ch/) and standardized via UniProt (https://www.uniprot.org/). Disease targets for MASH were retrieved from the GeneCards database (https://www.genecards.org/) using “NASH” as the keyword for human genes and deduplicated. The overlapping targets were imported into the STRING database (https://string-db.org/) to construct a protein-protein interaction (PPI) network with a confidence score threshold >0.700, after which isolated nodes were removed. The PPI network was visualized using Cytoscape 3.10.2 software, and core targets were identified by ranking based on degree values. The screening of core targets was performed according to the degree centrality metric. KEGG pathway enrichment of the overlapping targets was performed using the Metascape platform (https://metascape.org/). For molecular docking, the 3D structure of Rhein was downloaded from PubChem (https://pubchem.ncbi.nlm.nih.gov/). The crystal structure of EGFR (UniProt ID: P00533) was obtained from the Protein Data Bank (PDB) (https://www.rcsb.org/; PDB ID: 1M17). Docking simulations were carried out using CB-Dock2 (http://clab.labshare.cn/cb-dock2/), and the pose with the lowest (most negative) docking score was selected as the optimal binding conformation.

### Dual-Luciferase reporter assay

To evaluate the transcriptional activation activity of PPARα, AML12 cells were seeded into 24-well plates and transfected when cell confluence reached 70%–80%. Co-transfection was performed using BeyoFection™ Lipo8000 transfection reagent (# C0533, Beyotime, Shanghai, China). Each well received a total of 0.5 μg of DNA (Reporter plasmid pPPARα-TA-Luc 0.4878 μg and internal control plasmid pRL-TK 0.0122 μg) at a mass ratio of 40:1, together with 1 μL of Lipo8000 reagent. Six hours post-transfection, the transfection mixture was removed and replaced with DMEM/F-12 complete medium containing 10% FBS, followed by drug treatments as indicated: DMSO control group (final concentration <0.1%), Rhein treatment group (40 μM), PI3K inhibitor group (40 μM Rhein +20 μM LY294002), and GW7647 positive control group (100 nM GW7647). After 24 h of treatment, the culture medium was aspirated, and 100 μL of cell lysis buffer was added to each well. Cells were lysed with gentle shaking for 15 min at room temperature. Luciferase activities were measured using a Dual-Luciferase Reporter Assay Kit (# 11405ES60, Yeasen, Shanghai, China). Specifically, 20 μL of cell lysate was transferred to an opaque white 96-well plate, followed by the addition of 50 μL of Luciferase Assay Reagent II (LAR II) to measure firefly luciferase activity using a multimode microplate reader. Subsequently, 50 μL of Stop & Glo Reagent was added to quench the firefly reaction and simultaneously initiate the Renilla luciferase reaction, and the Renilla luciferase activity was measured immediately. The firefly luciferase activity of each well was normalized to the Renilla luciferase activity (Firefly/Renilla ratio). The relative luciferase activity was then calculated by normalizing the Firefly/Renilla ratio of each treatment group to that of the DMSO control group.

### Statistical analysis

In this study, statistical analyses of all data were performed using Graphpad Prism 8.0 software. Quantitative data were presented as mean ± standard deviation. Differences between two groups were analyzed using Student’s t-test, while differences among multiple groups were compared using one-way ANOVA. All experiments were repeated three times, and a P-value <0.05 was considered statistically significant.

## Results

### Rhein ameliorates MASH in a dose-dependent manner in mice

**FIGURE 1:**
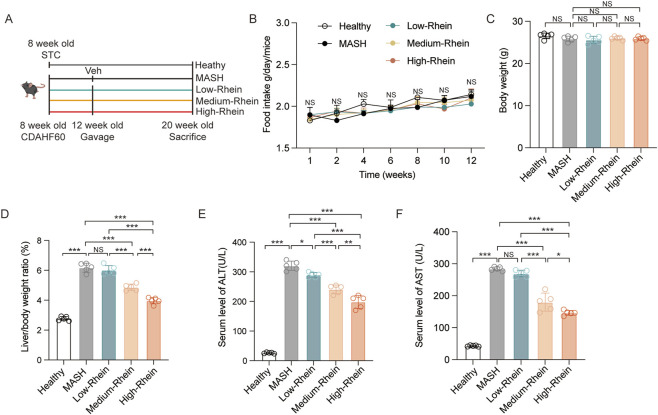
Rhein alleviates hepatic steatosis and injury in CDAHFD-induced MASH mice. **(A)** Schematic experimental design of the MASH mouse model and Rhein treatment. Male C57BL/6 mice (8 weeks old) were initially fed a CDAHFD-60% fat diet for 4 weeks to induce MASH. Subsequently, mice were orally administered Rhein (low, medium, or high doses) via gavage daily for 8 weeks, while the CDAHFD-60% diet was maintained throughout the entire 12-week induction period. Mice were euthanized for analysis at 20 weeks of age **(B)** Average daily food intake of mice from each group recorded throughout the 12-week experimental period (from 8 to 20 weeks of age) **(C,D)** Comparison of body weight and the liver-to-body weight ratio among groups **(E,F)** Serum levels of alanine aminotransferase (ALT) and aspartate aminotransferase (AST) indicating liver injury. Data are expressed as mean ± SD (n = 5 per group). Two-way ANOVA with Tukey’s test was used for Figure 1B. One-way ANOVA with Tukey’s test was used for Figures 1C–F. *P < 0.05, **P < 0.01, **P < 0.001.

To systematically evaluate the intervention effects of Rhein on metabolic dysfunction-associated steatohepatitis (MASH) and clarify its dose dependency, we established a mouse MASH model induced by feeding a choline-deficient, high-fat diet (CDAHFD). The experimental design is shown in Figure 1A. Mice were randomly divided into five groups: (1) Healthy control group (Healthy), fed a standard diet throughout; (2) MASH model group (Model), fed CDAHFD and administered the solvent vehicle via oral gavage; (3–5) Low- (20 mg/kg), Medium- (50 mg/kg), and High-dose (120 mg/kg) Rhein intervention groups (Low/Medium/High-Rhein). After 4 weeks of CDAHFD feeding to induce hepatic steatosis, these groups received 8 weeks of daily oral gavage with their respective doses of Rhein while continuing the CDAHFD.

First, we assessed basic metabolic parameters. The results showed no statistically significant difference in cumulative food intake among all groups over the 12-week experimental period (Figure 1B). At the experimental endpoint (week 12), no significant change in total body weight was observed among the groups (Figure 1C). However, significant alterations were noted in liver-related pathological indicators. The liver-to-body weight ratio was significantly higher in the MASH model group compared to the healthy control group. Notably, Rhein intervention dose-dependently reversed this trend. Compared to the model group, medium- and high-dose Rhein treatment significantly reduced the liver-to-body weight ratio, with the value in the high-dose group approaching that of the healthy control level (Figure 1D). In the assessment of liver function injury, serum levels of the hepatic injury markers alanine aminotransferase (ALT) and aspartate aminotransferase (AST) were markedly elevated in the MASH model group compared to the healthy controls. Rhein treatment demonstrated a clear protective effect, dose-dependently and significantly reducing serum ALT and AST levels (Figures 1E,F). Furthermore, we analyzed the serum lipid profile. As expected, serum TG and TC levels were significantly lower in the MASH model group than in the healthy control group, which may reflect the complex relationship between hepatic lipid metabolism dysregulation and systemic circulation. However, Rhein intervention at all doses did not significantly alter serum TG and TC concentrations in MASH mice (Supplementary Figure S1A,B).

### Rhein ameliorates MASH by enhancing fatty acid oxidation and suppressing inflammation

**FIGURE 2:**
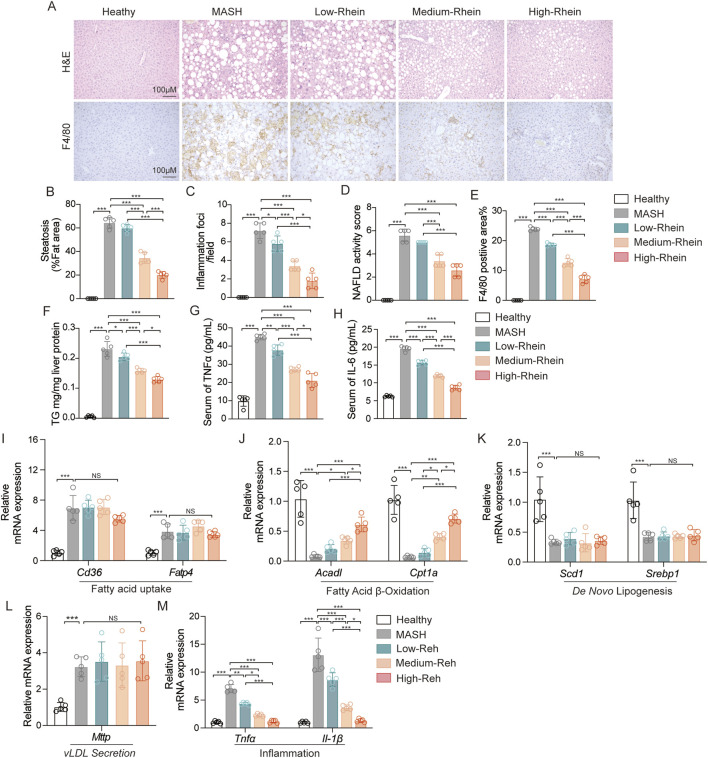
Rhein ameliorates MASH by enhancing fatty acid oxidation and suppressing inflammation. **(A)** Representative images of hematoxylin and eosin (H&E) staining and F4/80 immunohistochemical staining of liver sections from Healthy, Model, and Rhein-treated groups (Low, Medium, and High doses). Scale bar, 100 μm **(B,C)** Quantitative analysis of hepatic steatosis area **(B)** and inflammatory foci **(C)** based on H&E staining **(D)** Non-alcoholic steatohepatitis (NASH) activity score calculated according to standard histological criteria **(E)** Quantification of F4/80-positive area, indicating hepatic macrophage infiltration **(F)** Hepatic triglyceride (TG) content measured by biochemical assay **(G,H)** Serum levels of pro-inflammatory cytokines TNF-α **(G)** and IL-6 **(H,I–L)** Relative mRNA expression levels of genes involved in fatty acid uptake (*Cd36*, *Fatp4*), *de novo* lipogenesis (*Scd1*, *Srebp1*), and lipoprotein secretion (*Mttp*) in liver tissue **(J)** Relative mRNA expression levels of fatty acid β-oxidation–related genes *Acadl* and *Cpt1a* **(M)** Relative mRNA expression levels of inflammatory cytokines *Tnf-α* and *Il-1β* in liver tissue. Data are presented as means ± SD (n = 5 per group). One-way ANOVA with Tukey’s test was used for Figures 2B–M. *P < 0.05, **P < 0.01, ***P < 0.001.

Histopathological (Figure 2A) analysis of the liver revealed that, compared to the normal control group, the model group exhibited typical MASH pathological features, including significant hepatocellular steatosis (Figure 2B) and extensive inflammatory cell infiltration (Figure 2C). The comprehensive NASH activity score was significantly higher than the 5-point pathological diagnostic threshold (Figure 2D). After intervention with different doses of Rhein, the area of hepatic steatosis and the number of inflammatory foci showed a dose-dependent and significant reduction compared to the model group (Figures 2B,C). The NASH score also decreased significantly (Figure 2D), indicating that Rhein effectively reversed the core histological lesions of MASH. To quantitatively assess the degree of inflammatory cell infiltration in the liver, we performed immunohistochemical staining for the macrophage-specific marker F4/80 (Figure 2A). Quantitative analysis showed that the percentage of F4/80-positive area in the liver of the model group was significantly elevated (>20%), while in all Rhein treatment groups, especially the medium- and high-dose groups, this positive area was dose-dependently and significantly reduced (Figure 2E). Concurrently, hepatic biochemical analysis indicated a significant accumulation of triglyceride (TG) content in the liver tissue of the model group, and Rhein treatment dose-dependently lowered the intrahepatic TG levels (Figure 2F). This result was highly consistent with the alleviation of steatosis observed in H&E staining. At the systemic inflammation level, serum levels of the pro-inflammatory cytokines tumor necrosis factor-α (TNF-α) and interleukin-6 (IL-6) were significantly elevated in the model group compared to the control group. After Rhein intervention, the serum concentrations of both cytokines were significantly suppressed in a dose-dependent manner (Figures 2G,H). This result was consistent with the alleviation of local hepatic inflammation (Figures 2A,C,E).

To preliminarily elucidate the molecular mechanisms by which Rhein improves hepatic lipid accumulation and inflammation, we examined the mRNA expression of key metabolic and inflammation-related genes in the liver via qRT-PCR. Within the lipid metabolism pathways, Rhein treatment did not significantly alter the expression levels of genes related to fatty acid uptake (*Cd36*, *Fatp4*), lipogenesis (*Scd1, Srebp1c*), or lipoprotein secretion (*Mttp*) (Figures 2I,K,L). However, the results clearly demonstrated that Rhein significantly upregulated the expression of *Acadl* and *Cpt1a*, key genes responsible for fatty acid β-oxidation (Figure 2J). In the inflammatory pathways, Rhein treatment also significantly downregulated the mRNA levels of the local hepatic inflammation marker genes *Tnf-α* and *Il-1β* (Figure 2M).

In summary, these changes in the gene expression profile suggest that the mechanism by which Rhein alleviates hepatic steatosis in MASH mice may not involve the inhibition of lipid uptake or synthesis, but rather is primarily achieved by enhancing the hepatic capacity for fatty acid oxidation. Its anti-inflammatory effect appears to be closely associated with the direct inhibition of the activation of intrinsic hepatic inflammatory signaling pathways.

### EGFR identified as a potential key target of rhein in MASH by network pharmacology

**FIGURE 3:**
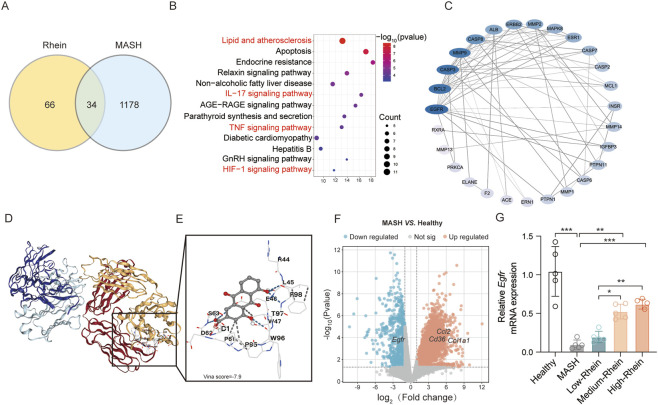
Identification of EGFR as a key target of Rhein in MASH through network pharmacology, molecular docking, and transcriptomic validation. **(A)** Venn diagram showing the overlap between predicted Rhein targets and MASH-related targets, yielding 34 common genes **(B)** KEGG pathway enrichment analysis of the overlapping targets, with the top enriched pathways ranked by −log10 (P value) **(C)** Protein–protein interaction (PPI) network constructed using the STRING database; node size and color intensity represent degree centrality, with EGFR identified as the core hub gene **(D)** Three-dimensional molecular docking model showing the binding conformation of Rhein within the active pocket of EGFR **(E)** Two-dimensional interaction diagram illustrating key amino acid residues involved in Rhein–EGFR binding **(F)** Analysis of *Egfr* mRNA expression in liver tissues from control and MASH model mice based on the GEO dataset GSE179322 **(G)** Relative mRNA expression levels of *Egfr* in liver tissues of Healthy, Model, and Rhein-treated mice as determined by qRT-PCR. Data are presented as means ± SD. One-way ANOVA with Tukey’s test was used for Figure 3G. *P < 0.05, **P < 0.01.

To elucidate the mechanism by which Rhein ameliorates MASH, we integrated network pharmacology predictions with experimental validation. Through Venn diagram analysis, 34 common targets were identified from 100 predicted targets of Rhein and 1,212 MASH-related targets retrieved from the GeneCards database (Figure 3A). These overlapping targets were submitted to the Metascape platform for KEGG pathway enrichment analysis. The results revealed significant enrichment in 75 pathways (P < 0.05). As shown in Figure 3B, the top 13 pathways ranked by P-value primarily involved lipid metabolism and atherosclerosis, inflammation and apoptosis pathways (e.g., TNFα, IL-17 and Hif-1 signaling pathways). Notably, lipid metabolism-related pathways were the most prominently enriched, aligning with the core pathological features of MASH and consistent with our earlier phenotypic findings that Rhein upregulates fatty acid oxidation genes (*Acadl, Cpt1a*) while downregulating pro-inflammatory genes (*Tnf-α, Il-1β*) (Figure 2). To identify key regulatory hubs within the protein-protein interaction network, the 34 overlapping targets were imported into the STRING database to construct a network, with isolated nodes removed. Network topology analysis indicated that the epidermal growth factor receptor (EGFR) possessed the highest degree centrality, positioning it at the core of the interaction network (Figure 3C). This suggests EGFR may be a key protein mediating the effects of Rhein. Subsequently, we assessed the binding potential between Rhein and EGFR through molecular docking. Typically, a binding energy below −5.0 kcal/mol indicates favorable binding activity (Saeed et al., 2017a; Saeed et al., 2017b). The docking simulation results showed that Rhein exhibits potential strong interaction with the EGFR active site in molecular docking, with a predicted binding energy of −7.9 kcal/mol (Figures 3D,E). This value is well below the common threshold, suggesting a potent and stable interaction between the two.

Finally, to verify the expression changes of EGFR during MASH progression and the regulatory effect of Rhein, we analyzed publicly available transcriptomic data from the GEO database (GSE179322). The analysis revealed a significant downregulation of *Egfr* mRNA expression in the livers of MASH model mice compared to the control group (Figure 3F). Consistent with this, in our established MASH mouse model, Rhein treatment dose-dependently reversed the decline in hepatic *Egfr* expression, restoring it to near-normal levels (Figure 3G). This series of coherent evidence—from bioinformatic prediction to computational simulation validation, and further to *in vivo* experimental validation—indicates that EGFR is a potential key target through which Rhein ameliorates MASH.

### Rhein reduces lipid accumulation and inflammation in hepatocytes *in vitro*

**FIGURE 4:**
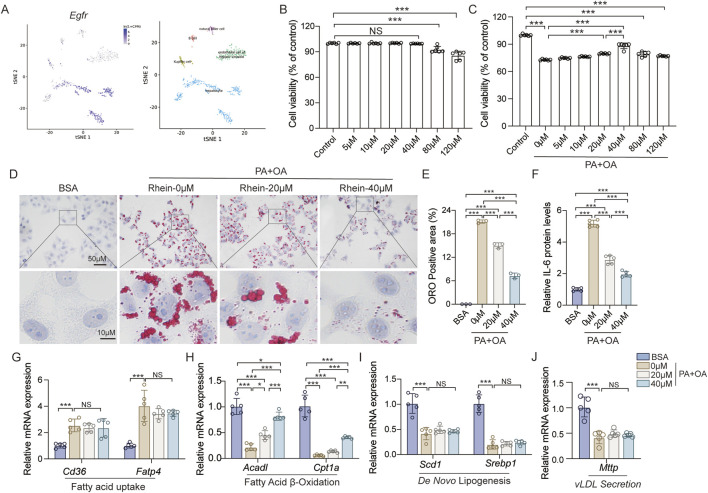
Rhein alleviates lipid accumulation and inflammatory responses in hepatocytes *in vitro.* **(A)** Expression profile of *Egfr* across major liver cell types analyzed from the Tabula Muris database, showing predominant expression in hepatocytes **(B)** Cell viability of AML12 hepatocytes treated with increasing concentrations of Rhein, as determined by the CCK-8 assay (n = 6 per group) **(C)** Protective effect of Rhein on PA/OA-induced lipotoxicity in AML12 cells assessed by CCK-8 assay (n = 6 per group) **(D)** Representative images of Oil Red O (ORO) staining showing intracellular lipid droplets in control, PA/OA-treated, and Rhein-treated AML12 cells. Scale bar, 50 μm (n = 3 per group) **(E)** Quantification of ORO-positive area reflecting intracellular lipid accumulation **(F)** IL-6 levels in cell culture supernatants measured by ELISA (n = 5 per group) **(G–J)** Relative mRNA expression levels of genes involved in fatty acid uptake (*Cd36*, *Fatp4*), lipogenesis (*Scd1*, *Srebp1*), and lipoprotein secretion (*Mttp*) (n = 5 per group) **(H)** Relative mRNA expression levels of fatty acid β-oxidation-related genes *Acadl* and *Cpt1a* (n = 5 per group). Data are presented as mean ± SD from at least three independent experiments. One-way ANOVA with Tukey’s test was used for Figures 4B,C,E–J. *P < 0.05, **P < 0.01, ***P < 0.001.

To elucidate the cellular mechanism of Rhein, we first analyzed the expression profile of *Egfr* across major liver cell types using the Tabula Muris database. The results showed that *Egfr* is predominantly highly expressed in hepatocytes, with minimal expression detected in Kupffer cells and endothelial cells (Figure 4A). Based on this finding, we selected the mouse hepatocyte line AML12 for subsequent *in vitro* mechanistic studies. The cytotoxicity of Rhein on AML12 cells was first assessed via the CCK-8 assay. The results indicated that Rhein demonstrated no significant cytotoxicity at concentrations up to 40 µM (Figure 4B), confirming its favorable safety profile *in vitro*. Subsequently, a lipotoxicity and inflammation model was established by treating cells with a palmitic acid/oleic acid (PA/OA) mixture to mimic the pathological microenvironment of MASH. In this model, Rhein treatment concentration-dependently improved cell viability, with the optimal effect observed at 40 µM (Figure 4C). Therefore, 20 μM and 40 µM were selected as the medium and high doses, respectively, for subsequent *in vitro* experiments. ORO staining revealed a significant increase in intracellular lipid droplets in the PA/OA model group. Rhein treatment concentration-dependently reduced this lipid accumulation, with the high-dose group showing a particularly pronounced effect (Figures 4D,E). Concurrently, measurement of the inflammatory cytokine IL-6 in the cell culture supernatant showed that Rhein concentration-dependently inhibited the PA/OA-induced increase in IL-6 secretion (Figure 4F). These results consistently demonstrate that Rhein effectively alleviates lipid accumulation and inflammation in hepatocytes *in vitro*, which aligns with the phenotypic improvements observed in our *in vivo* mouse experiments (Figures 2A,G,H).

To further investigate the underlying molecular mechanism, we examined the mRNA expression of key genes involved in lipid metabolism. The results showed that Rhein treatment did not significantly alter the expression of genes related to fatty acid uptake (*Cd36*, *Fatp4*), lipogenesis (*Scd1*, *Srebp1*), or lipoprotein secretion (*Mttp*) (Figures 4G,I,J). However, it significantly upregulated the expression of *Acadl* and *Cpt1a*, rate-limiting genes responsible for fatty acid β-oxidation (Figure 4H). This gene expression profile suggests that Rhein likely mitigates lipid accumulation primarily by enhancing the fatty acid oxidation capacity of hepatocytes, rather than by affecting lipid uptake, synthesis, or secretion pathways.

### EGFR integrity as a pivotal mediator of Rhein’s signaling

**FIGURE 5:**
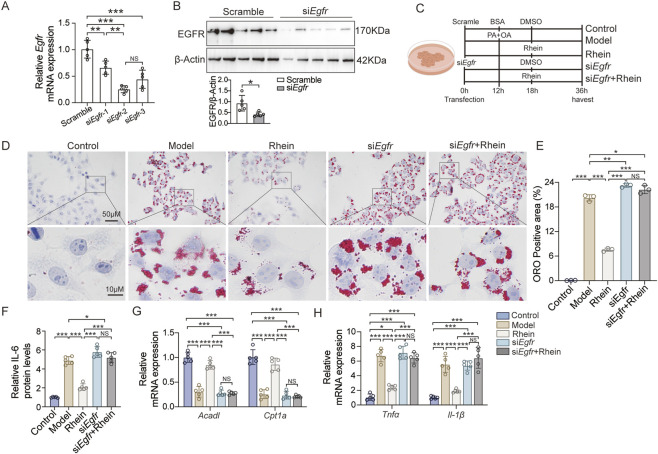
EGFR is essential for Rhein-mediated regulation of lipid metabolism and inflammatory responses in hepatocytes. **(A)** Screening and validation of siRNA sequences targeting *Egfr* in AML12 cells at the mRNA level by qRT-PCR (n = 5 per group) **(B)** Western blot analysis confirming efficient knockdown of EGFR protein expression by si*Egfr* (n = 5 per group) **(C)** Schematic illustration of the experimental design for *in vitro* EGFR loss-of-function studies **(D)** Representative Oil Red O (ORO) staining images showing intracellular lipid accumulation in Control, PA/OA model, PA/OA+ Rhein, PA/OA+ si*Egfr,* and PA/OA+ si*Egfr* + Rhein groups. Scale bar, 50 μm (n = 3 per group) **(E)** Quantitative analysis of ORO-positive area **(F)** IL-6 levels in cell culture supernatants measured by ELISA **(G)** Relative mRNA expression levels of fatty acid β-oxidation–related genes (*Acadl*, *Cpt1a*) (n = 5 per group) **(H)** Relative mRNA expression levels of pro-inflammatory cytokines (*Tnf-α*, *Il-1β*) (n = 5 per group). Data are presented as means ± SD from at least three independent experiments. Student’s t-test was used for Figure 5B. One-way ANOVA with Tukey’s test was used for Figures 5A,E–H. *P < 0.05, **P < 0.01, ***P < 0.001.

To elucidate the necessity of EGFR in Rhein’s regulation of hepatocyte lipid metabolism and inflammatory responses, we performed an *in vitro* loss-of-function validation. First, siRNA sequences targeting the mouse *Egfr* gene were designed and screened. Validation at the mRNA level showed that the siEGFR-2 sequence demonstrated a knockdown efficiency of approximately 80% (Figure 5A). This efficiency was further confirmed at the protein level by Western blot (Figure 5B), verifying the successful establishment of an AML12 cell model with low EGFR expression. Based on this model, five stringent experimental groups were established to dissect the core role of EGFR: Control group (Control); PA/OA model group (400 μM PA: 800 µM OA, Model); PA/OA+ Rhein group (40 µM Rhein, Rhein); PA/OA+ si*Egfr* group (si*Egfr*); PA/OA+ si*Egfr* + Rhein group (si*Egfr* + Rhein) (Figure 5C). Quantitative analysis of ORO staining revealed that Rhein treatment reduced intracellular lipid content in the model cells by approximately 62%. However, upon EGFR knockdown, the lipid-lowering effect of Rhein was completely abolished, with intracellular lipid levels showing no statistical difference from the untreated model group (Figures 5D,E). Concomitantly, the Rhein-induced reduction in the secretion of the pro-inflammatory cytokine IL-6 was also abrogated following EGFR silencing (Figure 5F). These results collectively demonstrate that the amelioration of both hepatocyte steatosis and inflammation by Rhein depends on intact EGFR signaling. qRT-PCR analysis showed that in control cells, Rhein increased the expression of the key fatty acid β-oxidation genes *Acadl* and *Cpt1a* by 2-3 fold (Figure 5G). However, this inductive effect of Rhein was completely lost in EGFR-knockdown cells. Conversely, regarding inflammation regulation, EGFR knockdown alone slightly upregulated the basal expression of *Tnf-α* and *Il-1β* (Figure 5H). More importantly, it completely reversed Rhein’s inhibitory effect on these two pro-inflammatory genes (Figure 5E). In the absence of EGFR, Rhein even exhibited a surprising trend of promoting inflammatory gene expression, which conversely underscores the protective and dominant role of EGFR signaling in this process.

### Rhein ameliorates hepatocyte metabolic dysfunction and inflammation via the EGFR/PI3K/AKT/PPARα signaling axis

To systematically elucidate the molecular mechanism by which Rhein improves lipid metabolism and inflammation in hepatocytes, and to validate the downstream pathways of its core predicted target EGFR based on network pharmacology, we focused on the classical EGFR/PI3K/AKT signaling axis and its potential downstream effector PPARα, conducting a series of gain-of-function and loss-of-function experiments.

**FIGURE 6:**
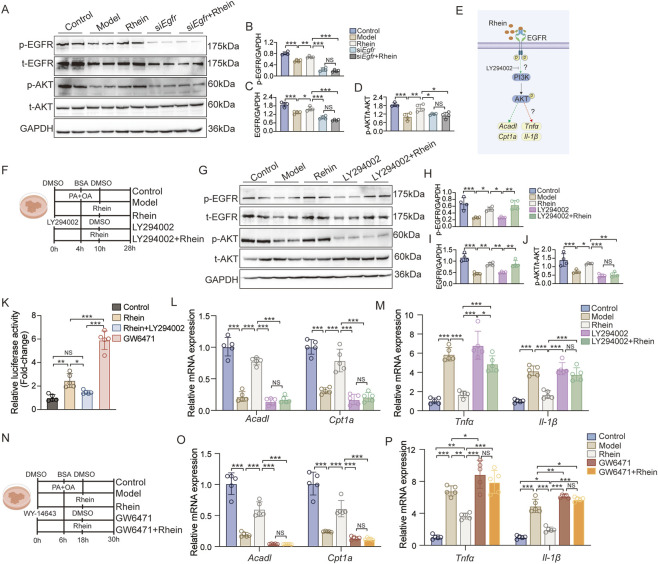
**(A)** Representative Western blot images showing the protein expression and phosphorylation levels of EGFR and AKT in AML12 cells under the indicated treatments (n = 4 per group). **(B–D)** Quantitative analysis of phosphorylated EGFR (p-EGFR), total EGFR, and phosphorylated AKT (p-AKT) normalized to corresponding total protein levels. **(E)** Schematic diagram illustrating the proposed EGFR/PI3K/AKT signaling pathway involved in Rhein action. **(F)** Experimental design for pharmacological inhibition of PI3K signaling using LY294002. **(G)** Representative Western blot images showing the effects of Rhein and PI3K inhibition on EGFR and AKT activation (n = 4 per group). **(H–J)** Quantitative analysis of p-EGFR, total EGFR, and p-AKT levels following PI3K inhibition. **(K)** Dual-luciferase reporter assay of PPARα transcriptional activity in AML12 cells under the indicated treatments (n = 5 per group). **(L,M)** Relative mRNA expression levels of fatty acid β-oxidation–related genes (Acadl, Cpt1a) and pro-inflammatory cytokines (Tnf-α, Il-1β) in AML12 cells treated with Rhein in the presence or absence of PI3K inhibition (n = 5 per group). **(N)** Experimental design for pharmacological inhibition of PPARα using the selective antagonist GW6471. **(O,P)** Relative mRNA expression levels of fatty acid β-oxidation–related genes (Acadl, Cpt1a) and pro-inflammatory cytokines (Tnf-α, Il-1β) following Rhein treatment with or without PPARα inhibition (n = 5 per group). Data are presented as means ± SD from at least three independent experiments. One-way ANOVA with Tukey's test was used for Figures 6B–D, 6H–J, 6K, 6L–M, 6O–P. *P < 0.05, **P < 0.01, ***P < 0.001.

First, we assessed the activation status of EGFR and its key downstream kinase AKT via Western blot analysis (Figure 6A). The results showed that in the PA/OA-induced lipotoxic and inflammatory model, the levels of phosphorylated EGFR (p-EGFR) and total EGFR protein were significantly downregulated compared to the normal control group (Figures 6B,C). Concurrently, the level of its downstream signaling node, phosphorylated AKT (p-AKT) (Figure 6D), was also synchronously reduced. This phenomenon suggests that the pathological microenvironment of MASH may suppress the basal activity of the EGFR/AKT signaling pathway. Following Rhein intervention, the expression of both p-EGFR and total EGFR protein significantly recovered. Importantly, the level of p-AKT was also restored alongside EGFR activation (Figures 6A–D). To establish the necessity of EGFR in this activation process, we utilized siRNA to stably knock down *Egfr* gene expression in AML12 cells. The results indicated that in the absence of EGFR expression, the upregulating effect of Rhein on p-AKT was completely blocked (Figures 6A–D). These data not only confirm that Rhein functions through EGFR to activate downstream signaling but also clarify that EGFR is an indispensable upstream receptor for Rhein to activate downstream AKT.

Based on the aforementioned findings, we hypothesized that PI3K, as the key bridging kinase connecting EGFR to AKT, is a necessary step for the downstream transmission of Rhein’s signal (a schematic of the signaling pathway is shown in Figure 6E). To functionally delineate the signaling hierarchy, we established five experimental groups: vehicle control, model (PA/OA), Rhein treatment, PI3K inhibitor (LY294002) alone, and Rhein combined with LY294002 (Figure 6F). Western blot analysis showed that, consistent with our prior findings (Figures 6A–D), Rhein treatment significantly increased the levels of both phosphorylated EGFR (p-EGFR) and total EGFR compared to the model group (Figures 6G–J). Notably, this upregulation was completely abrogated by co-treatment with the PI3K inhibitor LY294002, resulting in p-EGFR and total EGFR levels that showed no significant difference from the model group (Figures 6G–J). This result indicates that PI3K activity is required for Rhein-mediated EGFR/AKT signaling. Furthermore, while Rhein markedly increased AKT phosphorylation (p-AKT), this effect was fully abolished upon PI3K inhibition (Figures 6G,J). To directly assess PPARα transcriptional activity, we performed a dual-luciferase reporter assay in AML12 cells. As shown in Figure 6K, treatment with Rhein significantly increased relative luciferase activity by approximately 2.5-fold compared with the DMSO control group. Co-treatment with LY294002 completely abolished this increase, resulting in a relative activity similar to that of the control group. The PPARα agonist GW7647, used as a positive control, induced the highest luciferase activity (approximately 6-fold). Collectively, these data demonstrate that Rhein activates the AKT pathway through a sequential EGFR/PI3K-dependent mechanism. To further delineate the downstream pathway of the EGFR/AKT axis, we examined the expression of genes associated with fatty acid oxidation and inflammation. In line with our prior findings (Figures 4D–J), Rhein treatment significantly upregulated key fatty acid oxidation-related genes and downregulated pro-inflammatory genes compared to the model group (Figures 6L,M). However, when PI3K activity was inhibited by LY294002, these regulatory effects of Rhein on both fatty acid oxidation- and inflammation-related genes were completely abrogated, with expression levels showing no significant difference from those in the model group (Figures 6L,M). These results demonstrate that Rhein modulates downstream phenotypic outcomes, namely, by enhancing fatty acid oxidation and suppressing inflammation, through the EGFR/AKT signaling cascade.

Subsequently, we aimed to identify the direct effector molecule downstream of AKT responsible for regulating the expression of metabolic and inflammatory genes. The transcription factor PPARα is a major regulator of hepatic fatty acid β-oxidation and possesses anti-inflammatory properties (Kono et al., 2009; Toobian et al., 2021; Lin et al., 2022; Palenca et al., 2022). Furthermore, AKT signaling has been reported to positively influence PPARα transcriptional activity through various mechanisms, such as modulating its co-activator PGC-1α (Soyal et al., 2006; Fontecha-Barriuso et al., 2020; Qian L. et al., 2024). Therefore, we speculated that PPARα might serve as the terminal transcriptional regulatory hub of this pathway. To test this hypothesis, we employed the highly selective PPARα antagonist GW6471 for intervention (the experimental group design is shown in Figure 6N). By analyzing the expression profiles of lipid metabolism and inflammation-related genes via quantitative real-time PCR, we found that in the PA/OA model group, the expression of key fatty acid β-oxidation genes *Acadl* and *Cpt1a* was suppressed, while the expression of pro-inflammatory genes *Tnf-α* and *Il-1β* was significantly upregulated. Rhein treatment effectively reversed this gene expression profile. However, when PPARα activity was antagonized by GW6471, the inducing effect of Rhein on *Acadl* and *Cpt1a* expression, as well as its inhibitory effect on *Tnf-α* and *Il-1β* expression, were completely abolished (Figures 6O,P). This result functionally confirms that the transcriptional activity of PPARα is the final executing component through which Rhein achieves its regulation of metabolic and inflammatory gene expression.

Collectively, our experimental evidence indicates that Rhein ameliorates hepatocyte metabolic dysfunction and inflammation by targeting and activating EGFR on the cell membrane, thereby inducing its autophosphorylation. This signal is sequentially relayed through EGFR-dependent PI3K activation, which subsequently phosphorylates and activates AKT. The activated AKT then enhances the transcriptional activity of the nuclear receptor PPARα via downstream signaling. The potentiated PPARα mediates the transcriptional upregulation of rate-limiting genes in fatty acid β-oxidation while concurrently suppressing the expression of pro-inflammatory cytokine genes, likely through transrepression mechanisms. This coordinated transcriptional reprogramming restores lipid metabolic homeostasis and alleviates inflammatory responses in hepatocytes.

### The essential role of EGFR in mediating Rhein’s ameliorative effect on MASH *in vivo*

**FIGURE 7:**
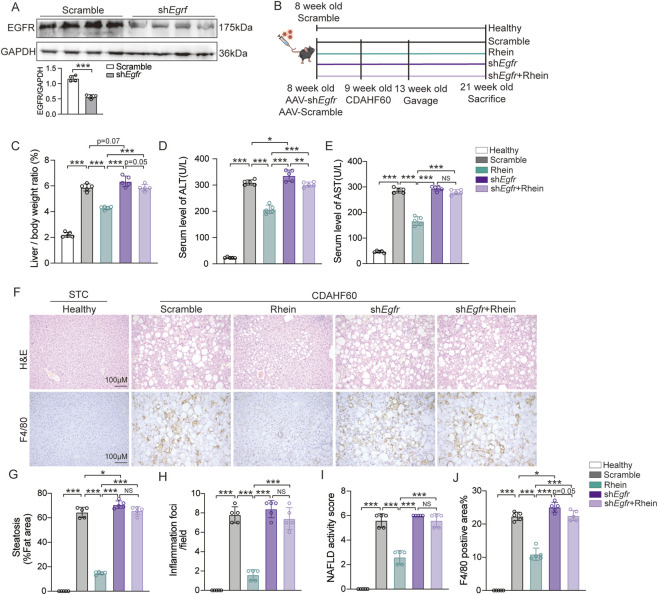
Hepatic EGFR is indispensable for the ameliorative effects of Rhein on MASH *in vivo.* **(A)** Western blot analysis confirming effective knockdown of EGFR protein expression in mouse liver following AAV8-sh*Egfr* administration (n = 4 per group) **(B)** Schematic illustration of the *in vivo* experimental design and timeline for AAV-mediated hepatic EGFR knockdown and Rhein treatment (n = 5 per group) **(C)** Liver-to-body weight ratio among different experimental groups **(D,E)** Serum levels of alanine aminotransferase (ALT) **(D)** and aspartate aminotransferase (AST) **(E,F)** Representative H&E-stained liver sections from each group. Scale bar, 100 μm **(G,H)** Quantitative analysis of hepatic steatosis area **(G)** and inflammatory foci **(H)** based on H&E staining **(I)** Non-alcoholic steatohepatitis (NASH) activity score **(J)** Representative immunohistochemical staining of F4/80 and quantification of F4/80-positive area, indicating hepatic macrophage infiltration. Scale bar, 100 μm. Data are presented as mean ± SD (n = 5 per group). Student’s t-test was used for Figure 7A. One-way ANOVA with Tukey’s test was used for Figures 7C–E,G–J. *P < 0.05, **P < 0.01, ***P < 0.001.

To verify at the *in vivo* level whether Rhein exerts its effects by interacting with EGFR, we constructed short hairpin RNAs (shRNAs) targeting the mouse *Egfr* gene. AAV8-sh*Egfr* or AAV8-scramble was delivered into mice via tail vein injection. Two weeks post-injection, Western blot analysis confirmed that AAV8-sh*Egfr* effectively reduced EGFR protein expression in the mouse liver (Figure 7A), indicating successful establishment of the *in vivo* knockdown model.

Based on this model, we designed five experimental groups (experimental timeline shown in Figure 7B): At 8 weeks of age, the control, model, and Rhein groups received AAV8-scramble, while the sh*Egfr* and sh*Egfr* + Rhein groups received AAV8-sh*Egfr*. After a 1-week recovery period, the control group was fed a standard diet, while the other four groups were fed a choline-deficient high-fat diet (CDAHFD) to induce MASH. After 4 weeks of CDAHFD feeding, the model and sh*Egfr* groups received daily oral gavage of saline, whereas the Rhein and “sh*Egfr* + Rhein” groups received Rhein (120 mg/kg/day) via gavage for 8 weeks, while maintaining the CDAHFD. Analysis revealed that compared to the normal control group, the liver-to-body weight ratio was significantly increased in the model group, and Rhein treatment significantly reduced this ratio, consistent with our previous findings (Figure 1C). However, when EGFR was knocked down (sh*Egfr* + Rhein group), Rhein’s effect on reducing the liver-to-body weight ratio was completely abolished, with the ratio showing no significant difference from the untreated model group (Figure 7C). Notably, EGFR knockdown alone (sh*Egfr* group) resulted in a liver-to-body weight ratio that was higher than that of the model group, showing a trend toward significance (p = 0.07) (Figure 7C). These results collectively indicate that EGFR not only possesses inherent hepatoprotective properties but is also an essential target for Rhein to exert its effect on reducing liver weight. Regarding liver function, serum levels of ALT and aspartate aminotransferase AST were significantly elevated in the model group, and Rhein treatment effectively reduced the levels of both enzymes. However, in the context of EGFR knockdown, Rhein’s ability to lower ALT (Figure 7D) and AST (Figure 7E) was similarly completely abolished. Histopathological assessment of the liver further corroborated these findings. H&E and F4/80 staining showed that Rhein treatment significantly alleviated hepatic steatosis (Figures 7F,G), reduced the number of inflammatory foci (Figures 7F,H), and decreased the F4/80-positive area (Figure 7J), thereby lowering the composite NASH pathological score (Figure 7I). However, when EGFR was knocked down, Rhein’s ameliorative effects on all these histopathological indices were completely blocked (Figures 7F–J).

The above *in vivo* experimental evidence demonstrates that EGFR is an indispensable target for Rhein to improve hepatic steatosis, inflammation, and injury in MASH mice. Rhein’s hepatoprotective effects strictly depend on the expression and function of EGFR in the liver.

## Discussion

This study systematically demonstrates through both *in vitro* and *in vivo* experiments that the naturally occurring anthraquinone compound Rhein effectively ameliorates MASH by coordinately activating the EGFR/AKT/PPARα signaling axis. This axis integrates metabolic regulation with anti-inflammatory actions, providing a novel mechanistic framework for understanding the hepatoprotective effects of Rhein and revealing the critical upstream regulatory role of EGFR in the pathophysiology of MASH.

Against the backdrop of the continuously rising global incidence of MASH and the current dilemma of having only one approved drug for treatment (Noureddin et al., 2024; Sanyal et al., 2024; Do et al., 2025), delving deeper into and scientifically elucidating the mechanisms of action of active components in traditional Chinese medicines will not only help advance drug development but also provide new perspectives for integrated traditional Chinese and Western medicine in treating metabolic liver diseases.

Our study demonstrates that Rhein treatment significantly alleviates hepatic steatosis, inflammatory cell infiltration, and reduces serum levels of liver injury markers in a dose-dependent manner, which is consistent with previously reported lipid-lowering and anti-inflammatory properties (Li et al., 2019; Huang et al., 2023). Notably, Rhein did not alter the systemic lipid profile or suppress genes related to fatty acid uptake or synthesis. Instead, it selectively upregulated key genes involved in fatty acid β-oxidation (*Acadl* and *Cpt1a*). This suggests that the primary mode of action by which Rhein ameliorates steatosis is through enhancing hepatic fatty acid oxidation, rather than modulating lipid influx or *de novo* synthesis. Integrating network pharmacology, molecular docking, and experimental validation, we identified EGFR as a key upstream regulator that mediates the effects of Rhein in MASH. EGFR exhibits the highest topological centrality within the protein-protein interaction network of the Rhein-MASH intersection targets. Molecular docking confirmed a high-affinity interaction between Rhein and EGFR (binding energy of −7.9 kcal/mol, significantly below the activity threshold of −5.0 kcal/mol) (Ivanova and Karelson, 2022; Terefe and Ghosh, 2022). Further investigations using *in vivo* and *in vitro* models revealed that Rhein treatment restored the downregulated expression and phosphorylation levels of EGFR under MASH conditions. Importantly, genetic knockdown of EGFR completely abrogated the beneficial effects of Rhein on lipid metabolism and inflammation, highlighting the necessity of EGFR in mediating Rhein’s actions (Wang et al., 2025). Notably, PI3K inhibition with LY294002 not only blocked AKT activation but also affected EGFR-related signals, suggesting the presence of feedback regulation or a more complex signaling network rather than a strictly linear EGFR–PI3K–AKT cascade. These observations indicate that EGFR and PI3K may regulate each other through reciprocal interactions, which warrants further investigation. Therefore, the present data support the involvement of the EGFR/PI3K/AKT signaling module in mediating the effects of Rhein, rather than definitive validation of a simple unidirectional signaling axis.

At the signaling level, this study systematically elucidates the sequential activation mechanism through which Rhein exerts its effects via the EGFR/AKT/PPARα axis. Under normal physiological conditions, EGFR and its downstream signaling play crucial roles in maintaining metabolic homeostasis in hepatocytes, whereas in the pathological context of MASH, the activity of this pathway is significantly suppressed. Our research reveals that Rhein functions through EGFR to restore its autophosphorylation level, thereby sequentially activating the phosphorylation of downstream PI3K and AKT. This process exhibits a strict dependency: the use of the PI3K-specific inhibitor LY294002 or knockdown of EGFR expression via siRNA completely blocks Rhein’s activation of AKT, confirming that the signal transduction proceeds sequentially along the EGFR/PI3K/AKT direction. The downstream transcription factor PPARα is identified as the terminal effector of this pathway: when the selective PPARα antagonist GW6471 is applied, Rhein’s induction of key fatty acid β-oxidation genes (*Acadl*, *Cpt1a*) and its inhibition of pro-inflammatory factors (*Tnf-α*, *Il-1β*) are completely reversed. This indicates that the transcriptional activity of PPARα is the final executing step through which Rhein achieves synchronized metabolic and inflammatory regulation.

These findings position Rhein as a multi-target regulator capable of synchronously modulating metabolic and inflammatory responses through a coherent signaling axis. Despite these advances, this study has certain limitations. First, it is important to note that our study did not directly evaluate fibrosis; therefore, the potential anti-fibrotic effects of Rhein remain to be investigated in future studies using longer-term MASH models or dedicated fibrosis assays. Second, the precise structural basis of the interaction between Rhein and EGFR requires further elucidation through crystallographic or mutagenesis studies. Third, the potential off-target effects and long-term safety of Rhein need to be evaluated in preclinical and clinical settings. Fourth, we did not reassess EGFR expression at the experimental endpoint, which is a limitation of the *in vivo* knockdown study. Finally, while this study focuses on hepatocytes, the holistic hepatoprotective effects of Rhein on non-parenchymal cells (such as Kupffer cells and hepatic stellate cells) remain to be explored. Additionally, studying whether Rhein can influence fibrosis reversal and hepatocellular carcinoma prevention in advanced MASH models would hold significant clinical relevance.

**FIGURE 8:**
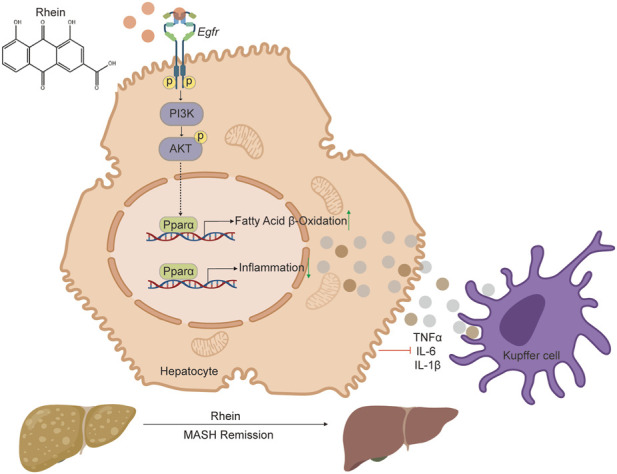
Schematic diagram illustrating the mechanism of Rhein in ameliorating metabolic dysfunction-associated steatohepatitis (MASH). Rhein specifically acts on the epidermal growth factor receptor (EGFR) on hepatocyte membranes, activating the downstream PI3K/AKT signaling pathway. The activated AKT translocates to the nucleus, where it enhances the transcriptional activity of peroxisome proliferator-activated receptor α (PPARα). This, in turn, upregulates the expression of key genes involved in fatty acid β-oxidation to promote lipid catabolism and concurrently suppresses the production of inflammatory cytokines such as TNF-α, IL-6, and IL-1β. This dual regulatory effect effectively alleviates lipid deposition and inflammatory responses in hepatocytes and indirectly inhibits the activation of hepatic macrophages (Kupffer cells). Consequently, at the whole-animal level, it leads to the resolution of hepatic steatosis, reduction of inflammatory infiltration, improvement in pathological scores, and ultimately contributes to the remission of MASH.

In summary, this study elucidates a novel mechanism by which Rhein ameliorates MASH through the synergistic effects of enhanced fatty acid oxidation and inflammation inhibition mediated by the EGFR/AKT/PPARα pathway. These findings demonstrate that Rhein improves steatosis and inflammation in MASH via the EGFR/AKT/PPARα pathway (Figure 8), suggesting the EGFR-PPARα axis as a potential therapeutic target. Whether Rhein affects fibrosis requires further investigation.

## Data Availability

The original contributions presented in the study are included in the article/Supplementary Material, further inquiries can be directed to the corresponding author.
