# Supplementary material for: Rhein ameliorates MASH via EGFR/AKT/PPARα-mediated coordinated regulation of metabolism and inflammation
Source: Front Pharmacol. 2026 Jun 23;17:1844294. doi: 10.3389/fphar.2026.1844294 (PMC13337408; doi:10.3389/fphar.2026.1844294)
Supplement: Supplementary file 1 [file Table1.docx]

Sequences of siRNAs and shRNA targeting mouse *Egfr*:

| Name | Sequence (5'-3') |
| --- | --- |
| si*Egfr*-1 | GAAGTGGTCCTTGGGAACTTGGAAA |
| si*Egfr*-2 | TGGAAATTACCTATGTGCAAAGGAA |
| si*Egfr*-3 | AACGTCTTTATGAGCAACATGTCAA |
| Scramble | GATGAGAGAGCAAGACGAATTCGAT |
| sh*Egfr* | TGGAAATTACCTATGTGCAAAGGAA |

The primer sequences used for quantitative real-time PCR are as follows：

| Name | Forward | Reverse |
| --- | --- | --- |
| m-Cd36 | ATGGGCTGTGATCGGAACTG | TTTGCCACGTCATCTGGGTTT |
| m-Fatp4 | ACTGTTCTCCAAGCTAGTGCT | GATGAAGACCCGGATGAAACG |
| m-Acadl | TCTTTTCCTCGGAGCATGACA | GACCTCTCTACTCACTTCTCCAG |
| m-Cpt1a | CTCCGCCTGAGCCATGAAG | CACCAGTGATGATGCCATTCT |
| m-Scd1 | TCTTCCTTATCATTGCCAACACCA | GCGTTGAGCACCAGAGTGTATCG |
| m-Srebp1 | TGACCCGGCTATTCC | CTGGGCTGAGCAATACAGTTC |
| m-Mttp | AATGCGGGTCAACAGAGAGG | CTGGCTCGTTTTCATAGGAGTAG |
| m-Tnfa | CCCTCACACTCAGATCATCTTCT | GCTACGACGTGGGCTACAG |
| m-Il-1b | CCGTGGACCTTCCAGGATGA | GGGAACGTCACACACCAGCA |
| m-Actin | GGCTGTATTCCCCTCCATCG | CCAGTTGGTAACAATGCCATGT |
