# Supplementary figures and images for: Rhein ameliorates MASH via EGFR/AKT/PPARα-mediated coordinated regulation of metabolism and inflammation
Source: Front Pharmacol. 2026 Jun 23;17:1844294. doi: 10.3389/fphar.2026.1844294 (PMC13337408; doi:10.3389/fphar.2026.1844294)

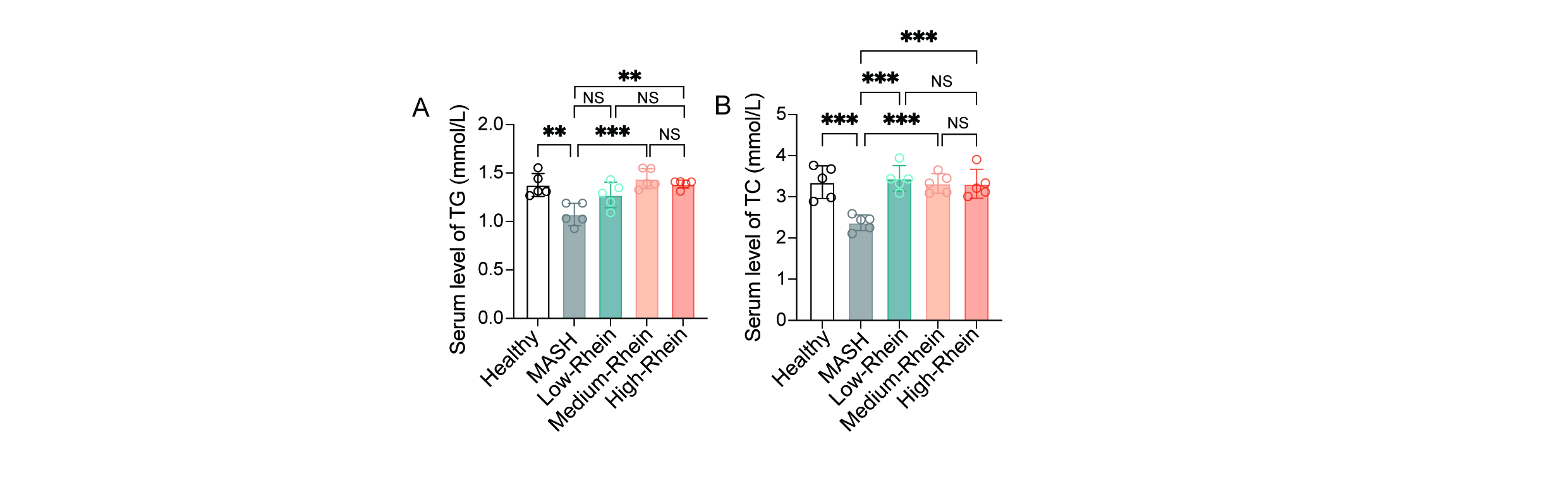

Supplement: Supplementary file 2 [file Image1.tif]
