# Supplementary material for: Rhein ameliorates MASH via EGFR/AKT/PPARα-mediated coordinated regulation of metabolism and inflammation
Source: Front Pharmacol. 2026 Jun 23;17:1844294. doi: 10.3389/fphar.2026.1844294 (PMC13337408; doi:10.3389/fphar.2026.1844294)
Supplement: Supplementary file 3 [file Table2.docx]

List of antibodies used for Western blot analysis:

| Antibody | Host Species | Dilution | Catalog No. | Vendor |
| --- | --- | --- | --- | --- |
| EGFR | Rabbit | 1:1000 | #4267 | Cell Signaling Technology |
| p-EGFR (Tyr1068) | Rabbit | 1:1000 | #3777 | Cell Signaling Technology |
| p-AKT (Ser473) | Rabbit | 1:1000 | #4060 | Cell Signaling Technology |
| AKT | Rabbit | 1:1000 | GB15689 | Servicebio |
| GAPDH | Rabbit | 1:5000 | GB11002 | Servicebio |
| Goat anti-Rabbit IgG | Goat | 1:2000 | GB23303 | Servicebio |
